# Long-term analysis of ventricular function in patients with symptomatic coronary disease who underwent on-pump or off-pump coronary artery bypass grafting

**DOI:** 10.1186/s13019-022-02069-1

**Published:** 2022-12-19

**Authors:** Rafael Rocha Mol Silva, Whady Hueb, Eduardo Gomes Lima, Paulo Cury Rezende, Paulo Rogério Soares, José Antonio Franchini Ramires, Roberto Kalil Filho

**Affiliations:** Instituto do Coração (InCor), Hospital das Clinicas HCFMUSP, Faculdade de Medicina, Universidade de Sao Paulo, Av. Dr. Eneas de Carvalho Aguiar 44 – AB 1 S114 Cerqueira Cesar, São Paulo, SP CEP – 05403-000 Brazil

**Keywords:** Coronary artery disease, Coronary artery bypass grafting, On-pump, Off-pump, Ventricular function

## Abstract

**Background:**

Systemic deleterious effects of cardiopulmonary bypass have been observed in the postprocedural period. Long-term assessment, including ventricular function (VF), is unclear. The objective of this study was to compare the change of left ventricular ejection fractions (LVEFs) during a long-term follow-up of coronary artery disease (CAD) patients who underwent off-pump (OPCAB) or on-pump coronary artery bypass grafting (ONCAB).

**Methods:**

This study is a prespecified analysis of the MASS III trial, which was a single-center and prospective study that enrolled stable CAD patients with preserved VF. The CAD patients in our study were randomized to OPCAB or ONCAB. A transthoracic echocardiogram was performed during follow-up and a LVEF value was obtained. The primary endpoint was the difference between the final LVEF and the baseline LVEF.

**Results:**

Of the 308 randomized patients, ventricular function were observed in 225 over a mean of 5.9 years of follow-up: 113 in the ONCAB group and 112 in the OPCAB group. Baseline characteristics were similar between the two groups, but there was a larger proportion of subjects with 3-vessel disease in the ONCAB group. There was no difference in the LVEF at the beginning (*P* = 0.08), but there was a slight decrease in the LVEF in the ONCAB and OPCAB groups (*P* < 0.001 in both groups) at 5.9 years. The decline was not significantly different between the two groups (delta of -6% for ONCAB and -5% for OPCAB; *P* = 0.78). In a multivariate analysis, myocardial infarction in the follow-up was a predictor of an LVEF < 40%.

**Conclusions:**

There was no difference in the long-term development of ventricular function between the surgical techniques, despite a decline in the LVEF in both groups.

*Trial registration* Clinical Trial Registration Information—URL: http://www.controlled-trials.com. Registration number: ISRCTN59539154. Date of first registration: 10/03/2008.

## Background

Left ventricular ejection fraction (LVEF) is the most important predictor of long-term survival in patients with coronary artery disease (CAD), regardless of the number of arteries affected, the degree of coronary stenosis, and the therapeutic strategies chosen [1, 2]. Coronary artery bypass grafting (CABG) is frequently performed and is the preferred method of treatment for patients with symptomatic multivessel disease [3] because it provides additional protection for the ischemic myocardium.

However, studies have reported changes in ventricular function after on-pump CABG (ONCAB), despite improvements in surgical techniques [4, 5]. The most common causes for these changes might be related to the use of cardiopulmonary bypass (CPB) and cardioplegic cardiac arrest that results in cardiac damage, myocardial stunning, systemic inflammation, bleeding diathesis, and multiorgan dysfunction [6].

However, off-pump CABG (OPCAB) has conflicting results despite its superiority in maintaining the beating heart and preserving cardiac function [7], and there is no large study that has evaluated the effect of CPB on LVEF as a long-term outcome.

Our objective was to compare LVEF in a long-term follow-up of symptomatic CAD patients who underwent ONCAB or OPCAB.

## Methods

### Study design

Protocol details were previously published [8]. In brief, the study included patients with angiographically documented proximal multivessel coronary stenosis > 70% by visual assessment, stable angina, and preserved ventricular function. Patients were enrolled and randomized if the surgeons agreed that either strategy could achieve revascularization. All angiograms were reviewed, and the surgical plan was documented before randomization. Patients were eligible if they were referred for isolated coronary bypass surgery for the first time, and an off-pump procedure was deemed technically feasible.

Patients were excluded if they required emergency or major concomitant surgery, unstable angina requiring emergency revascularization, ventricular aneurysm requiring repair, and an LVEF of less than 40%, previous stroke, peripheral vascular disease, or chronic renal insufficiency with an estimated creatinine clearance of less than 60 mL/min. Patients were also excluded if they were unable to provide written informed consent. In this prespecified analysis, we did not include patients who did not accomplish a new assessment of ventricular function by a transthoracic echocardiogram (ECHO).

The Ethics Committee of the Heart Institute of the University of São Paulo—Medical School, in São Paulo, Brazil, approved the trial, and all procedures were performed following the Helsinki Declaration. All subjects gave informed consent.

After giving consent, patients were randomized and the assignment was performed according to a computer-generated list of random permuted blocks that were unknown by the investigators. After randomization, patients were scheduled for the allotted treatment.

### Treatment protocols

In this study, all patients received goal-guided optimized clinical treatment. Coronary revascularization was optimized in accordance with current best practices. The surgeons had more than 20 years of experience and completed more than 100 procedures per year in both techniques. Stabilization devices were used during OPCAB to allow the safe construction of the anastomosis of the graft with the recipient artery. The patients were actively cooled to maintained a core temperature of at least 35 °C. Intracoronary shunts were not routinely used unless there was poor visibility, changes in the ST segment, or hemodynamic instability. CPB was established in a standardized manner using a membrane oxygenator, a roller pump, and without the use of cardiotomy suction. Patients were routinely cooled to 34 °C for operations with three grafts and 32 °C for four or more grafts. A cold cardioplegic crystalloid solution was anterograde administered directly to the root of the aorta and retrograde administered through the coronary sinus. For ONCAB, 1 g of intravenous hydrocortisone sodium succinate (SoluCortef®) was administered before anesthesia to reduce the inflammatory effects of CPB. A heparinization protocol of 300 U per kilogram for on-pump surgery and Protamine was used to reverse the effects of heparinization only in the on-pump patients.

### Study endpoints

The primary endpoint was the evolution of LVEF as a continuous variable in the form of the delta. This value was obtained by subtracting the final LVEF from the initial LVEF. Additionally, an LVEF less than or equal to 40% was attributed as a secondary outcome categorical variable in the dichotomized form.

### Assessment of ventricular function

Experienced echocardiographers from the same institution assessed left ventricular function on a two-dimensional echocardiogram with a transthoracic Doppler according to the techniques recommended by the specialty guidelines [9]. The first exam was performed before randomization, and a second exam was repeated throughout the follow-up. The LVEF was calculated by the end-diastolic volume (EDV) and the end stroke volume (ESV) using the following formula: LVEF = [(EDV) − (ESV)]/EDV. Simpson's method was used whenever there were changes in segmental contractility. The Teichholz method was used when there were no segmental changes.

### Follow-up

Patients were assessed every 6 months during the follow-up visits at the Heart Institute. Adverse events and clinical outcomes were considered from randomization. The primary outcome of the MASS III study was a composite of overall mortality, myocardial infarction, ischemic stroke, and additional revascularization.

### Statistical analysis

All data were analyzed on an intention-to-treat principle immediately after randomization, and values were expressed as the mean (± SD) or median (IQR). Dichotomous data were compared by the χ2 statistic or Fisher’s exact test. As evaluated through the Kolmogorov–Smirnov test, continuous variables that were not distributed normally were compared by the Mann–Whitney test. Student’s t test was used to compare continuous variables with a normal distribution. All reported probability values are 2-sided. Logistic regression analyses were used to identify variables associated with the development of ventricular dysfunction in the course, defined as an ejection fraction less than or equal to 40%. In addition, a multivariate analysis was performed to identify independent predictors of ventricular dysfunction in the follow-up. In this analysis, variables associated with an ejection fraction of less than or equal to 40% with marginal statistical significance (*P* < 0.20) in the univariate analysis were included in the model. Candidate variables included demographic, laboratory, clinical, surgical, and angiographic data. We used the backward stepwise method with the criterion of *P* < 0.05 to remain in the final model.

Interaction analyses evaluating the modification of the effect of surgical technique by the presence of clinical and surgical variables on the occurrence of ventricular dysfunction were also performed. The statistical significance of differences in treatment modalities on each endpoint was evaluated using the full population and a multiplicative interaction term. A probability value of *P* < 0.05 was considered statistically significant. These analyses were performed with SPSS, version 21.0 (SPSS, Inc.).

## Results

**Fig. 1:**
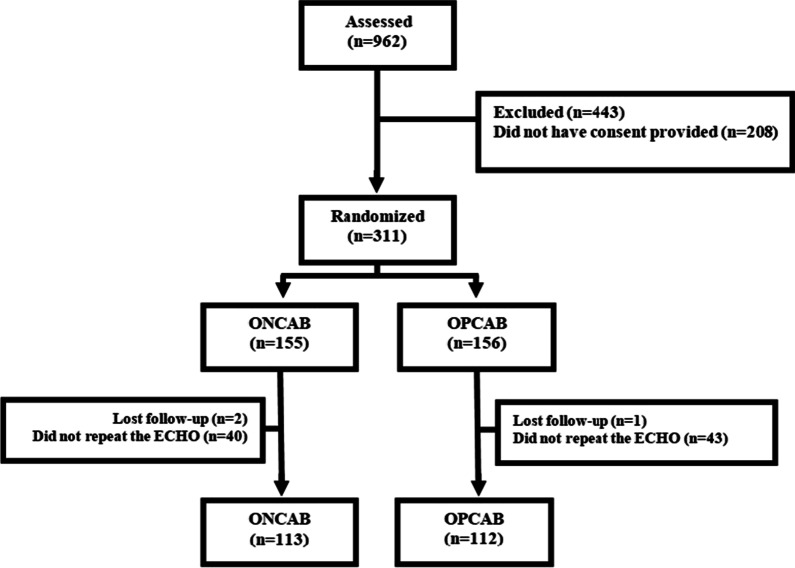
Number of patients assessed and randomized in this study. Follow-up: 5.9 ± 2.3 years. *ECHO* echocardiogram, *ONCAB* on-pump coronary artery bypass graft, *OPCAB* off-pump coronary artery bypass graft

Between March 2001 and March 2006, 962 patients with stable CAD and indications for revascularization were assessed, and 311 were randomized: 155 to the on-pump surgery group and 156 to the off-pump surgery group (Fig. 1).

Of those, left ventricular function of 225 patients were reassessed, 113 from the ONCAB group and 112 from the OPCAB group. The mean follow-up was 5.9 ± 2.3 years. Only three patients were lost to follow-up, and of the 83 who did not undergo a repeat ECHO, 29 died before the second echocardiographic evaluation. The remaining 54 did not undergo the second examination in the follow-up based on socioeconomic reasons related to the patient and/or the institution. As a result, a comparative analysis of the baseline variables was performed between the individuals who underwent a repeat echocardiogram and those who did not undergo a repeat echocardiogram at the end of the follow-up, and no significant difference was observed between the groups in terms of any of the variables, except age (with ECHO 59.4 vs. w/o ECHO 62.1; *P* = 0.01). Crossover occurred in three patients. Two of them due to hemodynamic disturbances and the other due to electrical instability.

The baseline demographic, clinical, and angiographic characteristics are summarized in Table 1. The mean age was 58.4 (± 0.8) years in the on-pump group and 60.5 (± 0.8) years in the off-pump group (*P* = 0.05). The prevalence of previous MI in this study was 44.6% in the ONCAB group and 46.9% in the OPCAB group, with no significant difference (*P* = 0.73). Although the treadmill test was positive in almost all patients, it was more frequently administered in the ONCAB group (*P* = 0.03). Similarly, three-vessel disease was more frequent in the ONCAB group (*P* = 0.01).

**Table 1:** Baseline characteristics

Characteristics	OPCAB (n = 112)	ONCAB (n = 113)	*P* value
*Demographic*			
Age (years)	60.5 ± 0.8	58.4 ± 0.8	0.05
Male sex (%)	73.2	77.9	0.41
*Medical*			
Previous MI (%)	44.6	46.9	0.73
Hypertension (%)	71.4	66.4	0.41
Diabetes mellitus (%)	38.4	32.7	0.37
Smoking (%)	22.3	31	0.33
Stable angina (%)	92.9	86.7	0.12
*Laboratory*			
Total cholesterol (mg/dL)	210.8 ± 4.4	216.2 ± 4.6	0.40
LDL-C (mg/dL)	130.2 ± 3.5	136.8 ± 4.2	0.22
HDL-C (mg/dL)	40.3 ± 0.9	40.4 ± 0.9	0.94
Triglycerides (mg/dL)	191.7 ± 11.5	182 ± 10.4	0.53
Glucose (mg/dL)	113.9 ± 2.6	119.6 ± 4.8	0.30
Creatinine (mg/dL)	1 0.9–1.2	1 0.9–1.2	0.99
*Functional*			
Positive treadmill test (%)	93.3	99	0.03
LVEF	70 (60–70)	66 (60–70)	0.08
*Angiographic*			
Three-vessel disease (%)	75.9	88.5	0.01
SYNTAX score	22 ± 0.7	23.9 ± 0.8	0.10
EURO score	0.86 (0.67–0.96)	0.85 (0.67–0.92)	0.95

Plus-minus values are means ± SE (standard error)

*HDL-C* high-density lipoprotein cholesterol, *LDL* low-density lipoprotein cholesterol, *LVEF* left ventricular ejection fraction, *MI* myocardial infarction, *ONCAB* on-pump coronary artery bypass grafting, *OPCAB* off-pump coronary artery bypass grafting

In addition, the SYNTAX score and EURO score showed no statistical difference between the groups.

Perioperative information is shown in Table 2. The median CPB time was 79 (IQR 69–94) minutes. The use of the left internal thoracic artery (LITA) had a greater tendency in the OPCAB group (99.1% versus 94.7%; *P* = 0.05). However, the use of the right internal mammary artery (OPCAB 14.3 versus ONCAB 32.1, *P* = 0.002) and the number of treated vessels were greater (OPCAB 2.6 ± 0.1 versus ONCAB 3.2 ± 0.1, *P* < 0.001) in the ONCAB group. The complete revascularization rate was 88.5% in the ONCAB group and 69.6% in the OPCAB group (*P* < 0.001). Finally, the CK-MB peak was 30 (IQR 18–58) mg/dL in the ONCAB group and 16.5 (IQR 12–25) mg/dL in the OPCAB group (*P* < 0.001).

**Table 2:** Periprocedural data

Variables	OPCAB (n = 112)	ONCAB (n = 113)	*P value*
Time on CPB (minutes)	NA	79 (69–94)	NA
LITA (%)	99.1	94.7	0.05
RITA (%)	14.3	32.1	0.002
Radial (%)	19.6	19.6	1.00
GEA (%)	6.3	4.5	0.55
Vein grafts (%)	76.8	81.4	0.39
Sequential grafts (%)	16.1	20.5	0.38
Grafts/patients (n)	2.6 ± 0.1	3.2 ± 0.1	< 0.001
CR (%)	69.6	88.5	0.003
CK-MB peak (mg/dL—IQR)	16.5 (12–25)	30 (18–58)	< 0.001

Plus-minus values are means ± SE (standard error)

*CPB* cardiopulmonary bypass, *CK-MB* creatine kinase-MB isoenzyme, *CR* complete revascularization, *GEA* gastroepiploic artery, *LITA* left internal thoracic artery, *RITA* right internal thoracic artery, *ONCAB* on-pump coronary artery bypass grafting, *OPCAB* off-pump coronary artery bypass grafting

**Fig. 2:**
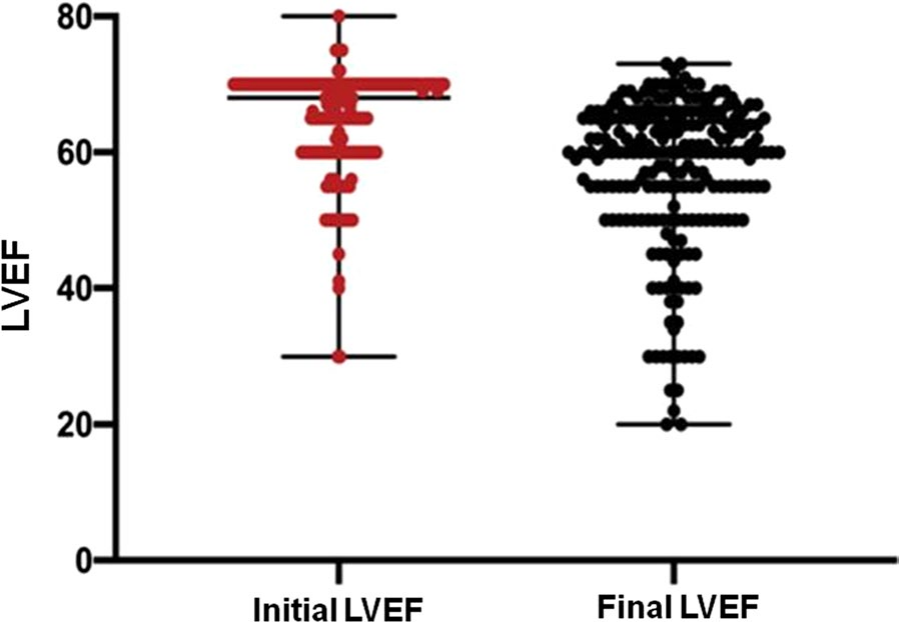
Comparison between initial and final LVEF in the general population

In the general population, there was a decline within the normal range in ventricular function, with a median LVEF initial of 68% and a final LVEF of 60% (*P* < 0.001), as shown in Fig. 2.

**Fig. 3:**
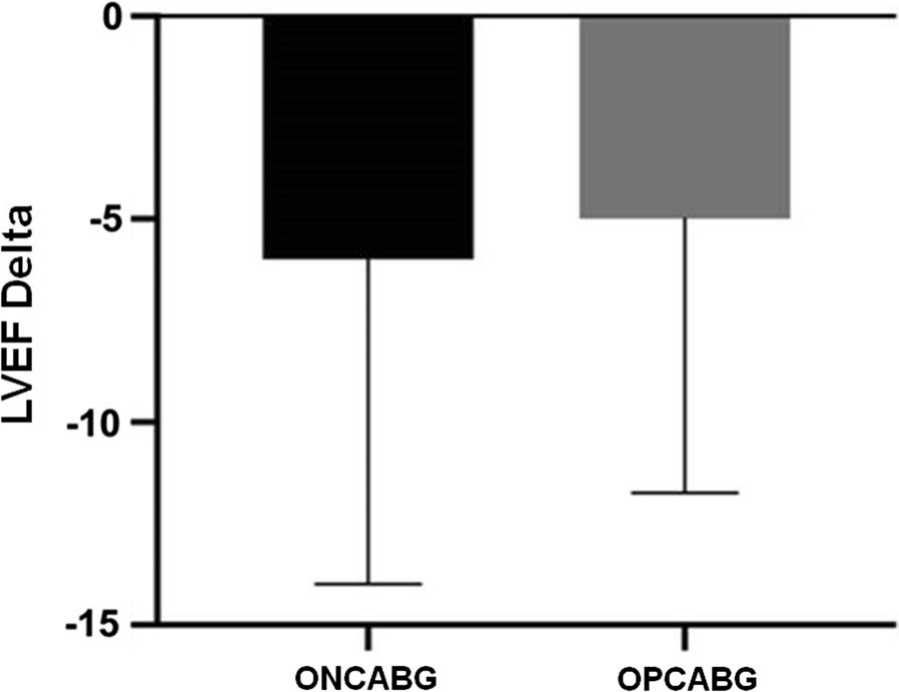
Comparison between the LVEF deltas according to the ONCAB and OPCAB groups

However, the comparison between the initial LVEF, 66% in ONCAB and 70% in OPCAB, and the final LVEF, 60% in ONCAB and 60% in OPCAB, as well as the decline in the delta form, did not show a significant difference, as shown in Fig. 3.

In addition, there were no significant differences in the occurrence of myocardial infarction (ONCAB 8.9% versus OPCAB 5.3%, *P* = 0.29) and further revascularization (ONCAB 8% versus OPCAB 6.3%, *P* = 0.61) between the CABG strategies.

We also analyzed the correlation of demographic, clinical, laboratory, functional, angiographic, and perioperative variables with the appearance of ventricular dysfunction. After the logistic regression with the multivariate analysis, we identified that only the peak of CK-MB (*P* = 0.008) and the occurrence of MI in both groups during follow-up (*P* = 0.02), regardless of the surgical technique (*P* = 0.81), were associated with an LVEF that was less than or equal to 40% (Table 3).

**Table 3:** Analyses of logistic regression for the identification of the predictors of ventricular dysfunction

Univariate	OR	95% CI	*P* value
*Demographic*			
Age*	0.96	0.91–1.01	0.13
Male sex	0.97	0.36–2.57	0.95
*Medical*			
Previous MI	3.47	1.39–8.70	0.008
Hypertension	1.28	0.53–3.06	0.57
Diabetes mellitus	1.86	0.71–4.86	0.20
Smoking	4.30	1.28–14.43	0.01
Stable angina	1.34	0.29–6.13	0.69
*Laboratory*			
Total cholesterol**	0.99	0.98–1.002	0.14
LDL-C**	0.98	0.97–1.001	0.07
HDL-C**	0.98	0.93–1.02	1.04
Creatinine**	0.88	0.43–1.80	0.73
*Angiographic*			
Three-vessel disease	1.15	0.37–3.56	0.80
*Surgical*			
ONCAB	0.90	0.39–2.07	0.81
IR	1.56	0.51–4.80	0.43
*Postoperative*			
CK-MB peak***	1.09	1.02–1.10	0.008
MI	4.29	1.35–13.61	0.01
*Multivariate*			
Postoperative			
CK-MB peak***	1.09	1.02–1.10	0.008
MI	4.64	1.21–17.69	0.02

*CK-MB* creatine kinase-MB isoenzyme, *HDL-C* *high-density lipoprotein cholesterol*, *LDL* *low-density lipoprotein cholesterol*, *LVEF* left ventricular ejection fraction, *MI* myocardial infarction, *ONCAB* on-pump coronary artery bypass grafting, *OPCAB* off-pump coronary artery bypass grafting

^*^Analyzed as a continuous variable for each year of life

^**^Analyzed as a continuous variable, for each 1 mg/dL

^***^Analyzed as a continuous variable, for every 10 ng/mL

**Fig. 4:**
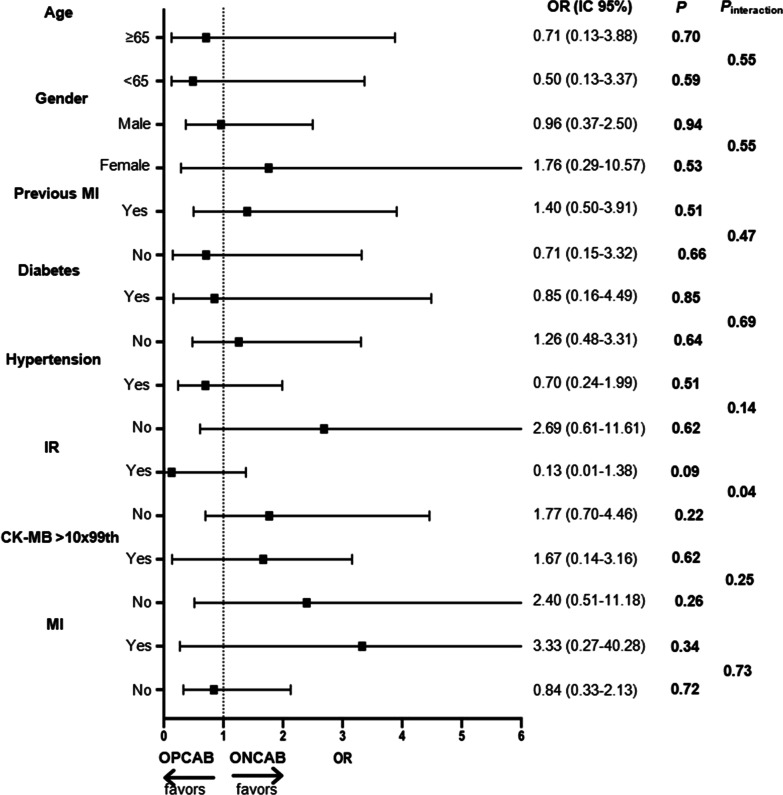
Interaction analysis between the surgical technique and selected variables in the evolution to ventricular dysfunction

Interaction analyses found that only incomplete revascularization (IR) showed a significant interaction (*P* = 0.04) with the surgical techniques regarding the occurrence of ventricular dysfunction (Fig. 4).

## Discussion

In the present study, the LVEF remained preserved in patients undergoing both operative techniques and did not show any difference in the long-term follow-up. Although the on-pump group showed a more significant release of necrosis biomarkers, even so, it did not result in impaired ventricular function. In this scenario, a significant decrease in the ejection fraction was observed in both groups when the release of necrosis biomarkers was accompanied by acute myocardial infarction.

This result was observed in individuals with multivessel coronary disease, in 95% of patients with documented ischemia, and in patients with similar demographics, clinical, laboratory, and ventricular function characteristics who underwent either surgery. Despite the groups’ similarities, the technical differences between the surgeries, especially regarding CPB, such as cannulation, aortic clamping, cardioplegic solution, and its systemic effects caused by the inflammatory response to the circuit, resulted in higher releases of myocardial necrosis biomarkers.

The release of myocardial necrosis biomarkers is a frequent occurrence after myocardial revascularization, and the pathophysiology underlying this phenomenon is complex and poorly understood [10]. Usually, cardiac injury is associated with direct myocardial trauma that results from manipulation of the heart, inadequate intraoperative cardiac protection, or microvascular events related to reperfusion and induced by oxygen free radical generation, which are potentially reversible conditions [11]. However, functional impairment presents when a cardiac injury meets the clinical criteria for myocardial infarction.

Data showed a slight decrease in the ejection fraction, which was observed in patients in both groups at the long-term follow-up. This decrease, within the normal range, can be explained by aging of the heart and the atherosclerosis of intramyocardial vessels. The heart undergoes complex changes during aging that affect the cellular composition, marked by a decrease in the absolute number of cardiomyocytes and exemplified by pathological alterations, including hypertrophy, altered left ventricular (LV) diastolic function, diminished LV systolic reverse capacity, increased arterial stiffness, and impaired endothelial function [12]

Achieving complete revascularization is challenging, and these challenges were present in this study. Although the surgical plan was created to balance the samples of the two groups before randomization, it was not implemented. The results showed greater completeness of revascularization in the patients in the on-pump group. On the other hand, considering the invasiveness of the procedure, patients in the on-pump group had longer intubation times, more significant bleeding and longer stays in the intensive care unit, without the added risk of impaired ventricular function. Even so, there was no significant change in LVEF over time.

Different operative techniques for surgical revascularization are complementary. The completeness of revascularization could compensate for the nonphysiological nature of CPB, and early hospital discharge compensates for the shortened surgical time. All of this without compromising LVEF.

To the best of our knowledge, our study was the only study that reassessed and compared systolic function between surgical techniques after 5 years. We performed an ECHO, an accessible, reproducible, and harmless diagnostic test for the patient, to estimate LVEF, a method of measuring systolic function validated from a diagnostic, therapeutic, and prognostic point of view in clinical practice [13].

There were some limitations. First, this analysis of measurements performed at 5.9 years did not include any patient who died before that time. Therefore, these results only applied to patients who were alive at the end, so it is possible that patients with a lower LVEF died before our evaluation. We performed an ECHO, which is an exam in which image acquisition is limited; however, intra- and interobserver variability, in terms of the method of measuring ventricular function, may present oscillations of magnitude that is equivalent to the median of the delta obtained at the end of the follow-up [13].

## Conclusions

There was no difference in the long-term evolution of ventricular function between the surgical techniques, despite the declines in the LVEF in both groups. The peak postoperative release of CK-MB and MI in the follow-up were the only independent predictors of ventricular dysfunction in this study.

## Data Availability

The datasets used and/or analyzed during the current study are available from the corresponding author on reasonable request.

## Abbreviations

CABG: Coronary artery bypass grafting
CAD: Coronary artery disease
CK-MB: Creatine kinase-MB isoenzyme
CPB: Cardiopulmonary bypass
CR: Complete revascularization
ECHO: Echocardiogram
EDV: End diastolic volume
ESV: End stroke volume
GEA: Gastroepiploic artery
HDL-C: High-density *lipoprotein cholesterol*
IR: Incomplete revascularization
LDL: *low-density lipoprotein cholesterol*
LITA: Left internal thoracic artery
LV: Left ventricular
LVEF: Left ventricular ejection fraction
MI: Myocardial infarction
OPCAB: Off-pump coronary artery bypass grafting
ONCAB: On-pump coronary artery bypass grafting
RITA: Right internal thoracic artery
SE: Standard error
VF: Ventricular function
